# Disorder robust, ultra-low power, continuous-wave four-wave mixing in a topological waveguide

**DOI:** 10.1515/nanoph-2024-0659

**Published:** 2025-04-08

**Authors:** Ju Won Choi, Byoung-Uk Sohn, George F.R. Chen, Hongwei Gao, William J. Mitchell, Doris K.T. Ng, Dawn T.H. Tan

**Affiliations:** Photonics Devices and System Group, 233793 Singapore University of Technology and Design, 8 Somapah Rd, Singapore, 487372, Singapore; NanoFab Facility, Electrical and Computer Engineering Department, University of California Santa Barbara, Santa Barbara, CA, 93106, USA; Institute of Microelectronics (IME), Agency for Science, Technology and Research (A*STAR), 2 Fusionopolis Way, Innovis #08-02, Singapore, 138634, Singapore

**Keywords:** four-wave mixing, topological devices, nonlinear integrated optics, ultra-silicon-rich nitride, CMOS

## Abstract

Four-wave mixing is a widely used nonlinear process for wavelength conversion, parametric amplification and signal regeneration in various Kerr devices, which enables wavelength-tunability and lower-power operation in compact optical systems. Here, we demonstrate low-power continuous-wave four-wave mixing in an ultra-silicon-rich nitride topological waveguide leveraging the strong confinement of the Su–Schrieffer–Heeger topological structure and ultra-silicon-rich nitride platform’s high Kerr nonlinearity and negligible nonlinear loss. We experimentally observe continuous-wave four-wave mixing at an ultra-low pump power of 510 µW, and wavelength tunability of 54 nm with on/off conversion efficiency of −57 dB at a pump power of 3 mW. We further investigate the efficiency of the four-wave mixing process when disorder is introduced into the Su–Schrieffer–Heeger waveguide array resulting in ±80 % randomness in the coupling coefficients. It is experimentally shown that similar conversion efficiencies are achieved in the presence and absence of disorder, indicating robustness against potential fabrication errors. We expect that this work can be applied to develop compact, tunable wavelength conversion systems operating at very low power levels which are robust against certain types of disorder.

## 1 Introduction

Four-wave mixing (FWM) has been extensively researched in various photonic structures such as waveguides, microring resonators, photonic crystal waveguides, and nanocavities [1], [2], [3], [4]. System miniaturization and chip-scale integration onto chips have enabled FWM to be generated at low pump powers [5]. The parametric processes at low pump power have been explored in microspheres [6], microtoid [7], microring resonator [2] and nanocavities [4] due to pump amplification occurring over enhanced light–matter interaction at the resonant condition and high quality factors. Nevertheless, these works are characterized by either fast response times or discontinuous wavelength tuning, owing to the specific spacing of resonances.

The connection between condensed matter physics and photonics was significantly reinforced when scientists proposed a photonic analog to the quantum Hall effect. This theoretical framework outlined the conditions required for unidirectional light propagation in photonic crystals involving time reversal symmetry breaking using magneto-optical materials in the presence of a magnetic field [8]. All-dielectric topological photonic devices on the other hand, leverage effective breaking of time-reversal symmetry. There has been significant recent progress in topological photonics, which has shown considerable promise in various applications including topological quantum light generation [9], [10], development of topological lasers, and implementation of photonic routing systems [11], [12], [13].

The application of topology emerged from research on topological insulators, where electrons can move along interfaces without energy loss, even when impurities are present. Following this principle, researchers can create special interfaces by engineering specific wavevector-space topologies. These interfaces support novel light states with unique properties. A key application is the development of unidirectional waveguides, where light can navigate around major defects without reflecting backwards [14].

Nonlinear phenomena has been theoretically studied in diverse topological systems by investigating edge states in the nonlinear Dirac model [15]. Experimental studies of nonlinear topological phenomena include switching between trivial and nontrivial state in a Floquet lattice of coupled waveguides by nonlinear effects [16], solitons generated by nonlinear effect in a photonic Floquet topological insulator [17], and combs implemented in a topological ring resonator [18].

In particular, the convergence of silicon photonics and topological photonics is transformative because it could unlock the promising commercial relevance of topological photonics based on the established numerous applications in silicon photonics [19], [20]. Topological photonic devices on silicon-based platforms can leverage CMOS manufacturing along with the implementation of integrated circuits. In addition, when studying nonlinear optics in topological photonic structures, silicon-based platforms may have high optical nonlinearities and/or high nonlinear figures of merit [21], making them excellent vessels for observations of nonlinear topological phenomena.

Photonic topological insulators guide light along topological boundaries using topological edge states. These topological waveguides utilize the zero modes created at the interface between two distinct topological domains within dimerized lattices. In the 1D Su–Schrieffer–Heeger (SSH) model, each unit cell possesses two sites, the topology of which is determined by the relative strengths of the intracell and intercell coupling. A domain wall arises at the boundary separating regions with different topological invariants, leading to the emergence of a topological mode, characterized as having its peak coincident with the boundary between the two regions. The topological mode is well localized to the domain wall and coupled light is strongly confined and propagates along the domain wall [22]. Protection against artifacts such as fabrication imperfections [9], [23], [24] and backscattering [25], [26], [27] have previously been demonstrated in a variety of topological systems. These unique capabilities enabled by topological light guiding are clear advantages that they have over total internal reflection -based light guiding in conventional photonic waveguides and have fuelled research in integrated topological photonics.

In this paper, we demonstrate topologically protected continuous-wave (CW) four-wave mixing (FWM) in a topological waveguide based on the Su–Schreiffer–Heeger model with a domain wall. We experimentally observe low-power parametric wavelength conversion in the topological SSH structure. Idler generation is observed at powers as low as 510 µW. We further achieve a tuning bandwidth of 54 nm for a pump power of 3 mW corresponding to an on/off conversion efficiency of −57 dB in the system. The pump power measured at the point of minimum conversion efficiency for CW FWM generation is the lowest among continuous-wavelength-tuning CW FWM in CMOS-compatible platforms. In addition, the efficiency of the FWM process is demonstrated to be similar when disorder in the SSH waveguide array is introduced. When the spacing between the SSH waveguide array possesses random disorder equivalent to 80 % in the coupling strengths between the waveguides (±41 nm), similar conversion efficiencies are recorded. This demonstration may facilitate potential applications in compact, low-power systems for wavelength conversion which are robust against disorder.

## 2 Materials and methods

Ultra-silicon-rich nitride (USRN) films are first deposited using chemical vapor deposition on SiO_2_ substrates. AFM measurements showed a smooth surface with a rms roughness of 0.27 nm for USRN films approximately 300 nm thickness. This surface roughness is comparable to that of the SiO_2_ substrate [28]. We use a thickness of 300 nm for USRN because the modal confinement for *H*=300 nm is better than for *H*=220 nm. The mode effective area (*A*_eff_) is almost at the minimum value for a USRN height of ∼300 nm for the width of 600 nm. *A*_eff_ is 0.46 μm^2^ for 300 nm and 0.5 μm^2^ for 220 nm. Therefore, based on the abovementioned reasons, we chose the USRN height of 300 nm rather than *H*=220 nm. The patterning of the SSH waveguide is performed using electron-beam lithography and etched using inductively coupled reactive ion etching. Lastly, plasma enhanced chemical vapor deposition is used to deposit 2 μm of SiO_2_ cladding. The topological photonic waveguide is structured as a coupled waveguide system that is dimerized based on the SSH model with a domain wall (Figure 2a). The waveguide is implemented on USRN, which is a CMOS-compatible platform possessing a large Kerr nonlinearity of *n*_2_ = 2.8×10^−13^ cm^2^/W and negligible nonlinear loss near the 1,550 nm wavelength [28], [29], [30], [31], [32], [33], [34], [35], [36]. The waveguide gap, *G*_w_, is narrower than *G*_v_ to achieve a non-trivial topology, where the intercell coupling strength (*w*) is greater than the intracell coupling strength (*v*). This system has already been demonstrated to exhibit good linear transmission properties due to strong light localization to the domain wall. *G*_w_ and *G*_v_ is set at 0.15 µm and 0.25 µm, respectively, to optimize strong localization with a coupling ratio of *v*/*w* = 0.17. The schematic of the device features a topological waveguide with a domain wall positioned at the center (boundary waveguide). In this design, 199 waveguides are utilized to ensure a sufficiently expansive transverse dimension in the topological system, especially when considering the localized boundary mode that spans 9 waveguides. Each waveguide has a cross-section of 600 nm (width) × 300 nm (height). The device’s length is 4 mm, which is 20 times longer than the coupling length of 200 µm for the long gap, a necessary condition to enable adequate coupling between waveguides during propagation and ensure sufficient nonlinear interaction [36].

The propagation dynamics of the electric field is described by the Schrödinger equation as follows:,

(1) $$i\frac{\partial E}{\partial z}+\frac{1}{2\beta_{0}}\nabla_{⊥}^{2}E+\frac{k_{0}^{2}n_{0}^{2}-\beta_{0}^{2}+2k_{0}^{2}n_{0}n_{2}{\left|E\right|}^{2}}{2\beta_{0}}E=0$$

**Figure 1:**
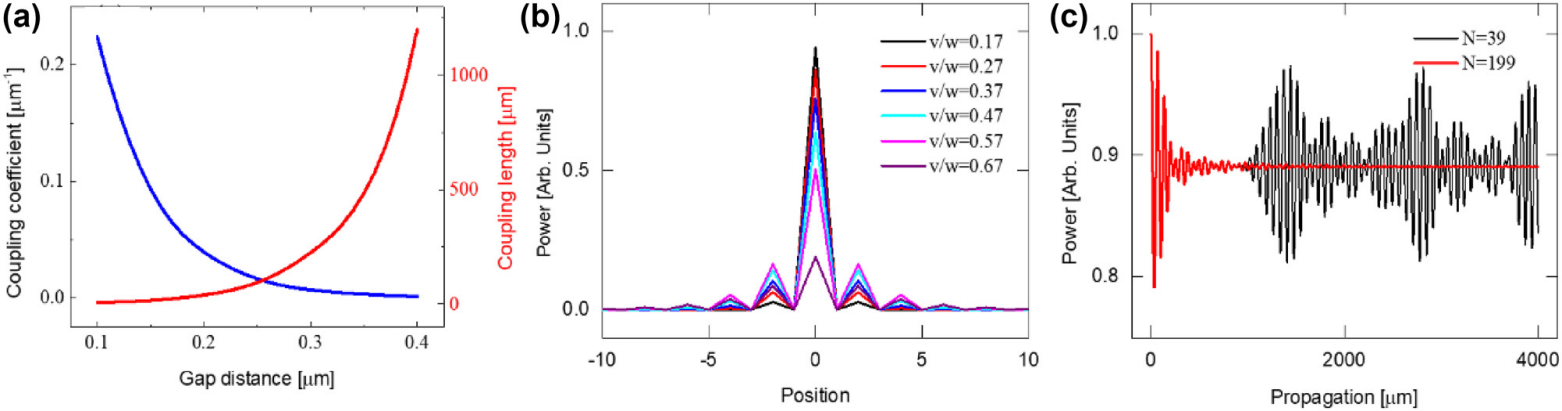
The theoretical propagation properties in the SSH waveguide. (a) The coupling coefficients (blue line) between two waveguides as a function of gap distance and the corresponding coupling length (red line). (b) The boundary modes for different v/w ratio (c) the power variation at the boundary waveguide for different number of waveguides [36].

The nonlinear Schrödinger equation can be represented by a Hamiltonian matrix for basis of single modes in single waveguides with each location. $K_{i,j}=\left\langle TE_{0i}\left|\frac{1}{2\beta_{0}}\nabla_{⊥}^{2}+\frac{k_{0}^{2}n_{0}^{2}-\beta_{0}^{2}+2k_{0}^{2}n_{0}n_{2}{\left|E\right|}^{2}}{2\beta_{0}}\right|TE_{0j}\right\rangle$ and $E=\sum_{i}A_{i}\left|TE_{0i}\right\rangle$ , where *i* represents the index of the waveguides, $\left|TE_{0\text{i}}\right\rangle$ and *A*_*i*_ is a TE single mode amplitude of fields for the *i*th waveguide. For the definition of design parameters, the Kerr nonlinear term is not considered here. Finally we get $i\frac{\partial A_{i}}{\partial z}+K_{i,j}A_{j}=0$ , *K*_*i*,*i*_=0 when *β*_0_ = *n*_*eff*_ *k*_0_, where *n*_*eff*_ is the effective refractive index for the boundary mode. The transverse derivative term vanishes by Gauss’ theorem. The off-diagonal terms, $K_{i,j\neq i}=-\frac{k_{0}^{2}}{2\beta_{0}}\left\langle T_{00,i}\left|n_{0}^{2}\right|T_{00,j\neq i}\right\rangle$ are the coupling coefficients. The coupling coefficient is calculated in Figure 1a for the different gap size between two waveguides with 600 nm×300 nm dimension.

For $K_{ij},i\&j\in \left\{-99,\ldots ,99\right\}$ , the Hamiltonian matrix is calculated to get the eigen mode for the boundary mode with zero eigen value. The zero mode is calculated in Figure 1b for different ratio between coupling coefficient (*w*) of short gap and that (*v*) of long gap. In Figure 1c, the output amplitude of *A*_0_ is calculated along to the propagation length at input initial value *A*_0_ = 1, *A*_*i*≠0_ = 0 for $K_{ij},i\&j\in \left\{-19,\ldots ,19\right\}$ (*N*=39) and $K_{ij},i\&j\in \left\{-99,\ldots ,99\right\}$ (*N*=199).

The coupling coefficient for the short gap, *G*_w_=0.15 µm is *w*=0.09. The coupling coefficient for the long gap, *G*_v_=0.25 µm is *v*=0.016. Under these conditions, the coupling length is 16 µm and 95 µm, respectively. The length of the sample (4 mm) is ∼40 times larger than the coupling length to ensure that the light undergoes sufficient multiples of the coupling length to exhibit topological effects. Similarly, the number of waveguides in the array (199) is large enough to remove the modulation effect which may occur over long propagation lengths as shown in Figure 1c; This modulation effect appears when the number of waveguides in the array is too small, as is illustrated for *N* = 39 (see Figure 1c).

The boundary mode is shown in Figure 1b for different *v*/*w* ratio. The mode becomes more tightly concentrated around the central waveguide when we increase *w* compared to *v*. It shows that the power is much more concentrated at the boundary when *v*/*w*=0.17. Consequently, we are using *v*/*w*=0.17 which has good confinement in the boundary. Therefore, we designed the SSH model to possess *G*_v_ = 0.25 μm and *G*_w_ = 0.15 μm which gives rise to *v*/*w*=0.17 and gives rise to the topological boundary at the center where the topological mode is highly concentrated to obtain the strong localization at the domain wall.

**Figure 2:**
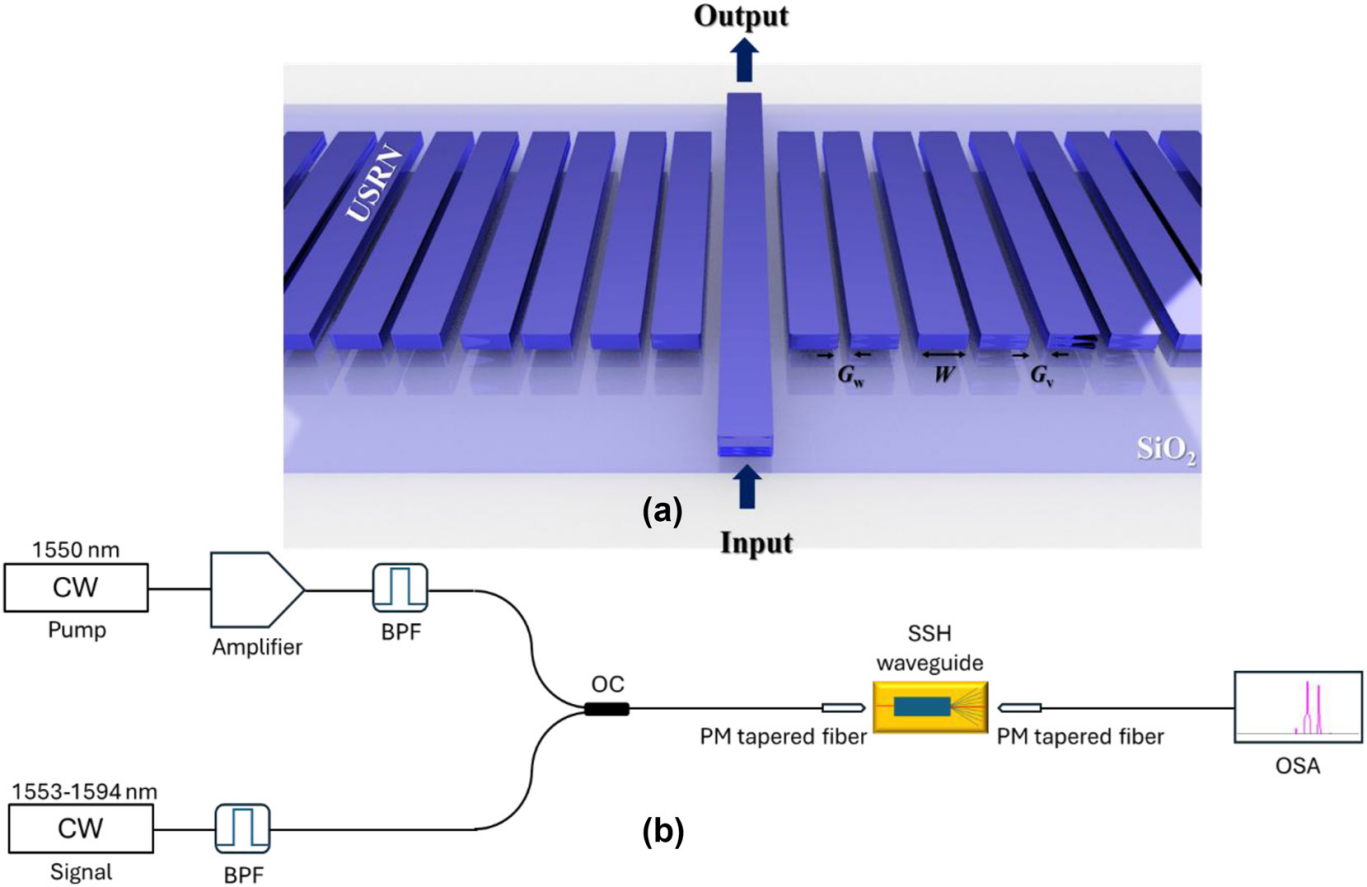
The design of the SSH waveguide and the experimental setup. (a) The schematic design of the topological SSH waveguide with a domain wall (The light comes in and out at the boundary waveguide). *W* is the width of the USRN waveguide. The values of the gaps between the waveguide are *G*_w_=0.15 µm and *G*_v_=0.25 µm. (b) The experimental setup for output spectral measurements. (CW: continuous-wave, BPF: bandpass filter, OC: 3 dB optical coupler, OSA: optical spectrum analyzer).

We first characterize FWM occurring in the SSH topological waveguide with no disorder. Figure 2b shows the experimental setup for measuring the output FWM spectra. A CW pump is first amplified using a polarization maintaining (PM) amplifier, prior to undergoing spectral filtering to remove the noise bands coming from the lasers themselves. The amplified and filtered CW pump is then combined with the CW signal using a 3 dB coupler and coupled into the boundary waveguide in the SSH structure via a tapered lensed fiber. The output from the device is coupled to a tapered lensed fiber and then the output spectra is analyzed using an optical spectrum analyzer. The pump wavelength is fixed at 1550 nm while the signal wavelength is tuned from 1553 nm to 1594 nm to observe the idlers. As a result of energy conservation, the frequency of the idlers is governed by the expression, *ω*_idler_ = 2*ω*_pump_ – *ω*_signal_. We note further that the FWM process in this case is degenerate, where two pump photons are annihilated to yield one photon at the signal and idler frequencies.

## 3 Results

**Figure 3:**
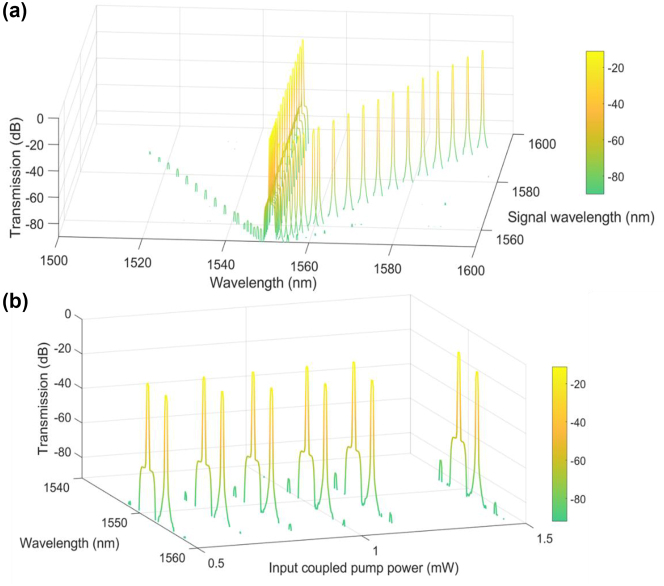
Output FWM spectra for (a) the signal wavelength varied from 1553 nm to 1594 nm at the input coupled pump power of 1.46 mW and for (b) the input coupled pump power varied from 0.51 mW to 1.46 mW at the signal wavelength of 1,554 nm. The pump wavelength is fixed at 1,550 nm.

The output FWM spectra are measured as shown in Figure 3. The pump wavelength is fixed at 1550 nm. Figure 3a shows the output FWM spectra when the signal wavelength is varied from 1553 nm to 1594 nm at the input coupled pump power of 1.46 mW. The generated idlers located in the blue side of the pump are obtained. The gap between idler wavelength and pump wavelength is wider when signal wavelength in the red side is more far from the pump because of energy and momentum conservation rules based on FWM. Small peaks located near and at the red side of the signal depict second idlers which can be generated when pump and signal wavelengths are close each other. In this case, the frequency of the generated idlers is governed by the expression, *ω*_idler_ = 2*ω*_signal_ – *ω*_pump_, indicating that the FWM process is degenerate at the signal wavelength – two signal photons are annihilated to generate one photon each at the pump and idler frequencies. The output FWM spectra are also observed when the input coupled pump power is varied from 0.51 mW to 1.46 mW at the signal wavelength of 1554 nm where the larger idler is observed as shown in Figure 3b. The power in the first and second idlers is observed to increase as pump power increases.

**Figure 4:**
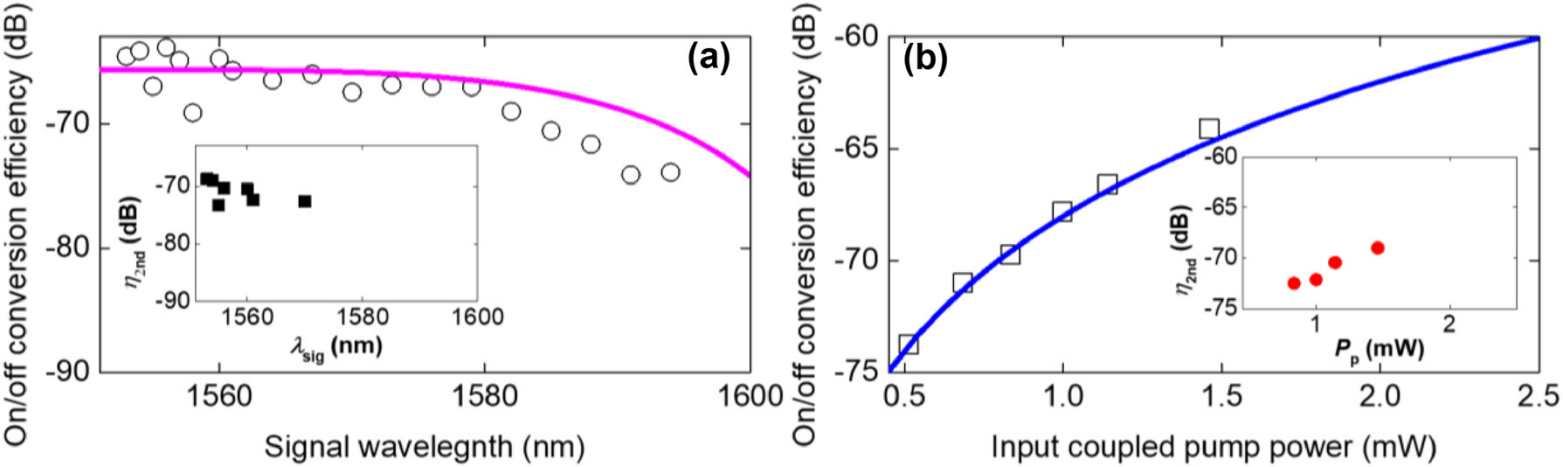
On/off conversion efficiencies in the SSH waveguide. (a) On/off conversion efficiency (empty circles) is obtained from the FWM spectra (Figure 3a) and is compared with theoretical curves (pink line). The inset of (a) shows the on/off conversion efficiency for second idlers (*η*_2nd_) as a function of signal wavelength (*λ*_sig_) (b) On/off conversion efficiency (empty squares) is obtained from the FWM spectra (Figure 3b) and is compared with theoretical curves (blue line). The inset shows the on/off conversion efficiency for second idlers (*η*_2nd_) as a function of pump power (*P*_p_).

Based on the measured spectra as shown in Figure 3, we calculated the on/off conversion efficiency as shown in Figure 4. The measured on/off conversion efficiency is defined as *P*_idler,out_/*P*_signal,out_, where *P*_idler,out_ is the output idler power measured using OSA and *P*_signal,out_ is the output signal power with the pump off.

The theoretical on/off conversion efficiency (CE) is described according to the equation [1], [34], [37],

(2) $$\text{On}/\text{off CE}={\left[\frac{\gamma P_{p}}{g}\times \sinh\left(gL_{\mathrm{eff}}\right)\right]}^{2}\cdot {\text{e}}^{\alpha \cdot L_{\mathrm{eff}}}$$

where $g=\sqrt{{\left(\gamma P_{p}\right)}^{2}-{\left(\frac{\left(2\gamma P_{p}+\beta_{2}\Omega^{2}\right)}{2}\right)}^{2}}$ . Here, *γ* is the nonlinear parameter and *P*_p_ is the input coupled pump power. *L*_eff_ is the effective length defined as $\frac{1}{\alpha }\left[1-e^{-\alpha \cdot L}\right]$ , where *α* and *L* are nonlinear parameter of 2 cm^−1^ and device length of 4 mm. *β*_2_ is the group velocity dispersion parameter and *Ω* is pump-signal frequency detuning of (*ω*_p_ – *ω*_s_) (*ω*_p_ = angular frequency at pump_,_ *ω*_s_=angular frequency at signal). We fix the pump wavelength at 1550 nm. We note that analysis using these equations has previously been compared with the coupled equations and shown to give similar results in the undepleted pump approximation [36]. The undepleted pump approximation in this case holds since the pump power is considerably higher in power than the signal and idler powers.

Figure 4a shows the experimental on/off CE (empty circles) as a function of signal wavelength when pump power is fixed at 1.46 mW obtained from the output FWM spectra (Figure 3a). The theoretical on/off CE as a function of signal wavelength for a pump power of 1.46 mW is calculated and found to be in good agreement with the experiment. The on/off CE is slightly decreases as the pump-signal frequency detuning is increases. This shows that the first idler can be generated with a wide bandwidth of 41 nm at a pump power as low as 1.46 mW. The inset of Figure 4a depicts the measured on/off CE for second idlers (*η*_2nd_) that is obtained from varying the signal wavelength (*λ*_sig_) from 1553 to 1570 nm. No second idlers are observed when the signal wavelength is > 1570 nm. *η*_2nd_ decreases slightly as the pump-signal wavelength detuning increases, a similar phenomenon as that observed for the on/off CE for first idlers at a fixed pump power.

Figure 4b shows the experimental on/off (empty squares) CE as a function of pump power when the signal wavelength is fixed at 1554 nm where higher conversion efficiency is obtained. Figure 4b is obtained from the output FWM spectra from Figure 3b. The measured on/off CE increases as pump power increases, and it agrees well the theoretical on/off CE (blue line) at the signal wavelength of 1554 nm. The inset of Figure 4b depicts the measured on/off CE for second idlers (*η*_2nd_) as a function of pump power (*P*_p_). *η*_2nd_ increases as pump power increases, a trend which is similar to that observed for the on/off CE for first idlers at a fixed signal wavelength. For the generation of the first idler, the governing equation is *ω*_first idler_ = 2*ω*_pump_ – *ω*_signal_. Two pump photons are annihilated to generate one photon at the signal wavelength and one photon at the first idler wavelength. For the generation of the second idler, the governing equation is *ω*_second idler_ = 2*ω*_signal_ – *ω*_pump_. Two signal photons are annihilated to generate one photon at the pump wavelength and one photon at the second idler wavelength. In both cases, the FWM conversion efficiency has a quadratic dependence on the first term on the right-hand side of the Eq. (2). Since the pump power is higher than the signal power, the FWM conversion efficiency will be higher for the first idler compared to the second idler. Second idlers are observed when the pump power is increased from 0.83 mW to 1.46 mW. This indicates that in our system, up to 2nd idlers can be observed when the pump power is as low as < 1 mW. 1st idler generation can be observed when the pump power is as low as 0.51 mW. These results suggest that our SSH system with strong localization of light to the domain wall facilitates interaction of the co-propagating pump and signal fields with the USRN material for efficient CW four-wave mixing even at small pump powers of < 1 mW. The topological waveguide can generate CW FWM satisfying both critical conditions of ultra-low pump power of 0.51 mW and wavelength-tunability (41 nm). This feature enables the demonstrated SSH waveguide system to be used towards compact, low-power frequency generation systems.

**Figure 5:**
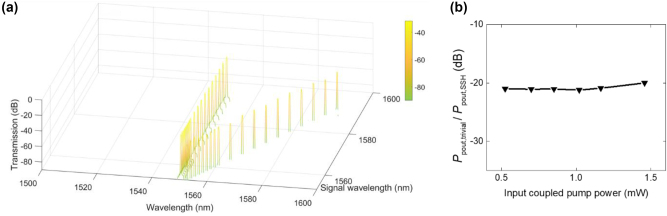
Optical properties in the trivial waveguide compared to the SSH waveguide. (a) Output spectra in non-topological (trivial) waveguide. Pump wavelength is 1,550 nm and signal wavelength is tuned from 1,553 nm to 1,594 nm. The pump power is 1.46 mW same as that in topological waveguide. (b) The ratio of output pump power in trivial case (*P*_pout, trivial_) to output pump power in topological case (*P*_pout, SSH_) as a function of input pump power.

To verify CW FWM in topological waveguide, we conducted the same experiment using a trivial (non-topological) waveguide that has a fixed gap of 0.25 µm between the waveguides in the array. This configuration prevents light from being localized in the boundary waveguide [36]. The same input pulse conditions were applied to the central waveguide. The output spectra are measured when the pump wavelength is fixed at 1550 nm while the signal wavelength is tuned from 1553 nm to 1594 nm for the pump power of 1.46 mW as shown in Figure 5a. There are no idlers observed regardless of pump-signal frequency detuning. In the trivial waveguide, where all elements are equidistant, light is not localized to the boundary waveguide but instead diffuses away from the input, similar to spatial diffraction. Consequently, lower output power is measured. The ratio of output pump power in trivial case (*P*_pout, trivial_) to output pump power in topological case (*P*_pout, SSH_) is obtained to > −20 dB which indicates that much higher insertion loss occurs in the trivial waveguide than topological waveguide as shown in Figure 5b.

**Figure 6:**
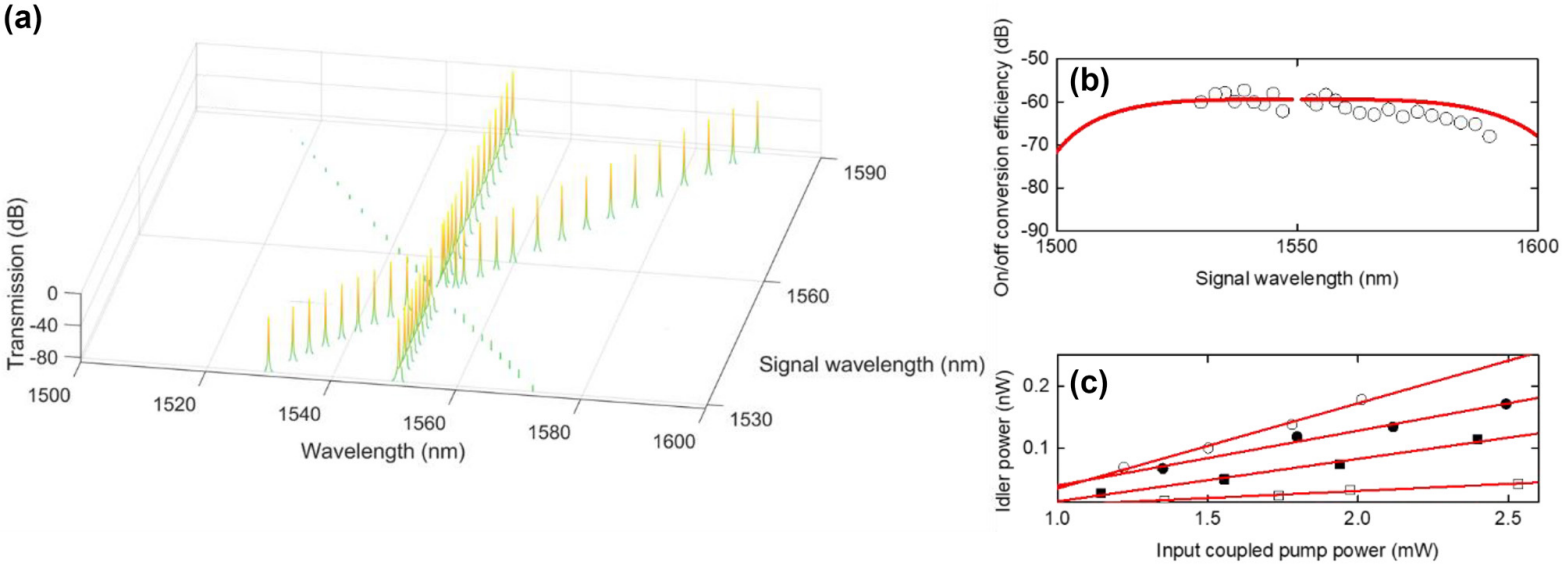
The FWM properties for an input power of up to 3mW in the SSH waveguide. (a) Output FWM spectra for the signal wavelength varied from 1,530 nm to 1,590 nm at the input coupled pump power of 3 mW. (b) On/off conversion efficiency (empty circles) is obtained from the FWM spectra and is compared with theoretical curves (red line). (c) Idler power as function of input pump power for the signal wavelength of 1,533 nm, 1,546 nm, 1,554 nm, and 1,578 nm. The idler power is linearly proportional to pump power, agreeing well with linear fit (red lines). The pump wavelength is fixed at 1,550 nm (◯) *λ*_sig_ = 1,533 nm, (⚫) *λ*_sig_ = 1,547 nm, (■) *λ*_sig_ = 1,554 nm and (□) *λ*_sig_ = 1,578 nm.

To obtain wider tuning bandwidth, a higher gain amplifier is used to increase the input CW pump power to 3 mW. The output FWM spectra is obtained by tuning the signal wavelength from 1,530 to 1,547 nm and 1,553–1,590 nm as shown in Figure 6a. It is experimentally observed that idlers can be generated regardless of the location of the signal wavelength whether it is on the blue or red side of the pump wavelength. The signal tuning range is limited due to the optical tunable bandpass filter helping to suppress noise of the CW source in both pump and signal lines. Based on the FWM spectra, the on/off conversion efficiency of the first idler is obtained to range from −57 dB to −68 dB having maximally 54 nm of signal tuning bandwidth as shown in Figure 6b. The experiment results also agree well with the theoretical conversion efficiency (red line). The measured first idler powers are measured for the different signal wavelengths of 1,533 nm, 1,546 nm, 1,554 nm, and 1,578 nm as shown in Figure 6c. The linear relation between idler power and pump power has a good agreement with the linear fits (red lines). It indicates that the FWM process as a function of pump power proceeds efficiently regardless of signal wavelength.

The conversion efficiency depends on the phase mismatch, Δ*k* = 2*γ*.*P*_p_ + *k*_s_ + *k*_i_ – 2*k*_p_, where *k*_p_, *k*_s_, and *k*_i_ are the pump, signal and idler wavenumbers. To get higher conversion efficiency, it is ideal for Δ*k* to be zero. In photonic waveguides where GVD varies slowly with wavelength, *k*_s_ + *k*_i_ – 2*k*_p_ is approximately equal to *β*_2·_(*ω*_p_ – *ω*_s_)^2^ for small pump-signal detuning. Based on the conversion efficiency equation in Eq. (2), we could plot the conversion efficiency as a function of signal wavelength to see how much bandwidth is tolerable. Theoretically, in our condition, the tuning bandwidth is achievable to ∼77 nm at 3 dB bandwidth for the operating maximum powers of 1.46 mW and 3 mW. Due to the limited tunability of CW laser and bandpass filter, our experimentally measurable bandwidth is limited to 54 nm. However, from theory, the device can cover the tuning bandwidth including the telecommunication C-band (1530–1565 nm) and a part of S- (1460–1530 nm) and L-bands (1565–1625 nm).

One of the key advantages of topological photonic structures is their robustness against artifacts such as fabrication imperfections. Our SSH device with a domain wall comprises 199 waveguides in the array, possessing gaps *G*_w_ and *G*_v_. During fabrication, imperfections could cause the gaps to deviate to different extents throughout the entire array resulting in fluctuations in the coupling coefficients. We design an additional SSH device with varying extents of randomness in the gaps. The device has the following extents of disorder in the gap, Δ*d* = ±41 nm, leading to 80 % randomness in effective coupling coefficients within the array. We study its performance for four-wave mixing compared to a device without disorder.

**Figure 7:**
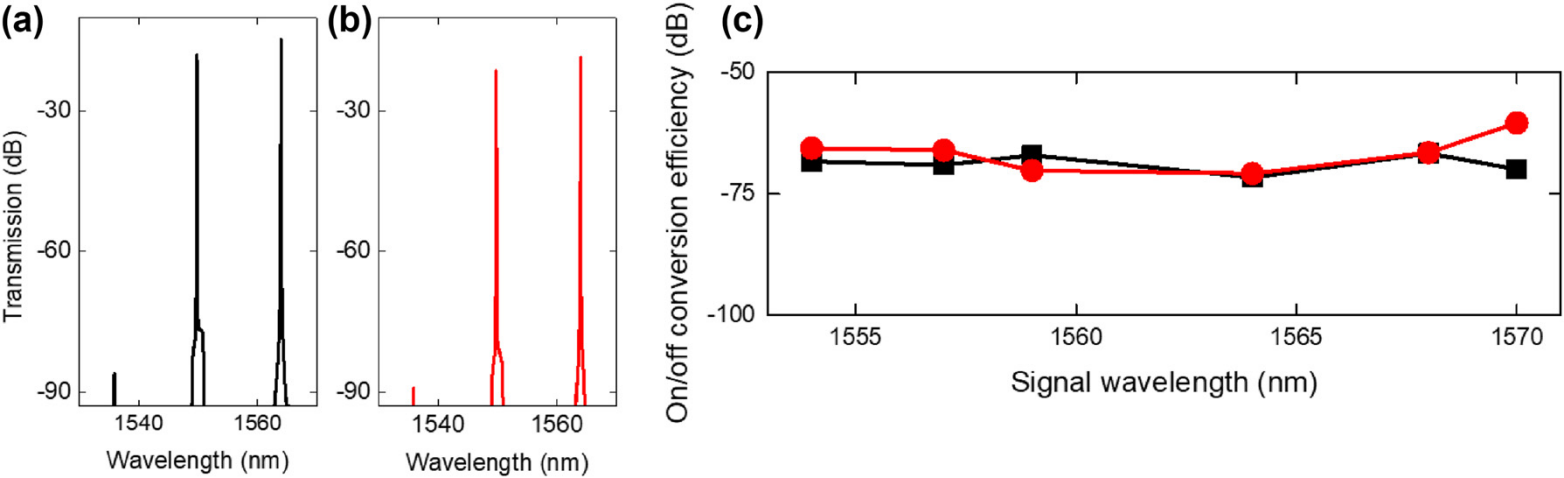
Output FWM spectra of SSH devices for a signal wavelength of 1,564 nm (a) without and (b) with disorder (Δ*d* = ±41 nm) in the coupling coefficients in the array. (c) On/off conversion efficiency of SSH devices without disorder (black squares) and disorder in the SSH array (Δ*d* = ±41 nm) (red circles) as a function of signal wavelength.

Figure 7 shows the comparison of on/off conversion efficiency of SSH devices without disorder in the gaps of the waveguide array (Δ*d* = 0 nm, same as the SSH device with *G*_w_ = 0.15 µm and *G*_v_ = 0.25 µm) and with disorder intentionally introduced (Δ*d* = ±41 nm) as a function of signal wavelength for the input coupled power of 1.4 mW. The result shows the conversion efficiency with and without disorder are similar for the tested signal wavelengths, indicating robustness to disorder in the gaps. Light continues to be well localized within the domain wall in the presence of disorder without becoming delocalized and leaking to the adjacent nearest neighbor waveguides, enabling very similar pump-signal photon interactions to occur for the devices with and without disorder. This unique attribute of the SSH device makes it tolerant to fabrication imperfections and showcases its robustness against randomness in the spacings of the waveguide array in achieving nonlinear parametric wavelength conversion.

The conversion efficiency at the lowest measured input pump power and tuning bandwidth for CW FWM in other CMOS-compatible waveguide platforms are compared to verify the effectiveness of generating CW FWM in terms of wavelength tuning and low power conversion, as shown in Table 1. Amongst the CW FWM in CMOS platforms, we could experimentally measure CW FWM at the lowest pump power (−2.92 dBm) as well as a relatively wide conversion bandwidth of 54 nm. Some waveguides with different platforms such as silicon and AlGaAs could achieve a larger conversion bandwidth, but they require longer waveguide lengths of a few cm or much higher pump power to generate CW FWM. In addition, resonators like the silicon coupled resonator waveguide and graphene-clad silicon nanocavity could achieve efficient CW FWM at the lower pump power, but the conversion is not continuous and occurs only at discrete wavelengths coincident with the discrete resonance properties of optical resonators and nanocavities [4], [47]. In the absence of slow light or resonance enhancements, only the photonic waveguide and SSH waveguide implemented in USRN could generate an observable FWM idler at pump powers < 1 mW. In both cases, the high Kerr nonlinearity in USRN facilitated strong pump-signal interactions for the conversion even at very low pump powers. It may also be inferred that the strong localization of the topological mode to the domain wall facilitates the interaction of the pump and signal photons with the USRN medium, thus facilitating CW FWM at very low pump powers of 0.5 mW, as well as observations of a conversion bandwidth of 54 nm. It also indicates that the robustness in conversion efficiency observed in the topological waveguide facilitates the CW FWM process even at small pump power. This attribute is useful for the implementation of wavelength conversion systems with low-power consumption with wide spectral tuning and high compactness.

**Table 1:** Minimum input pump power and wavelength tuning range for CW FWM in CMOS-compatible platforms (Experiment).

Platform	*L* (mm)^a^	Conversion efficiency at *P*_min_^b^	Maximum BW(nm)
Silicon WG^d^ [38]	15.8	−51 dB at <0.5 dBm	17
Graphene-silicon slow-light PCWG^e^ [39]	0.2	−28 dB at 12 dBm	17
AlGaAs WG^d^ [40]	25	−35 dB at 15 dBm	63.8^g^
Low-loss silicon nitride WG^d^ [41]	1,000	−50 dB at >20 dBm	2.7
Thick silicon WG^d^ [42]	350	−37 dB at >14 dBm	5
High index doped silica glass SWG^f^ [43]	500	−40 dB at 22 dBm	11
USRN WG^d^ [31]	7	−45 dB at −1 dBm	>90
Silicon-graphene strip WG^d^ [44]	3	−58 dB at 3 dBm	35
Low-loss silicon WG^d^ [45]	27	−21 dB at 16 dBm	25
Amorphous silicon carbide WG^d^ [46]	2.5	−55 dB at >10 dBm	>50
Our work	4	−73.7 dB at −2.92 dBm	54

^a^ *L* is interaction length. ^b^The lowest measured input coupled pump power. ^c^Maximum conversion bandwidth. ^d^WG: waveguide. ^e^PCWG: photonic crystal waveguide. ^f^SWG: spiral waveguide. ^g^Maximum BW for the length of 5 mm.

## 4 Discussion

The classical mechanism driving light confinement is the use of total internal reflection (TIR) which requires a core with a higher refractive index than its surrounding cladding. While TIR is lossless in principle, the materials themselves introduce absorption and scattering losses. Photonic bandgaps provide another avenue for light confinement and guiding even in lower refractive index media. This confines light within specific wavelength ranges using a complete two-dimensional photonic bandgap (PBG) in the cladding structure. The cladding’s PBG is created by a periodic array of air holes, which form a photonic crystal. The bandgap of a photonic crystal is caused by destructive interference of light reflecting between layers of high and low refractive index regions [48].

Each waveguide in our SSH modelled array has a higher core index than cladding index which utilizes TIR. Adding to TIR, the topological design also helps to strongly confine light to the domain wall.

For propagation in the *y*-direction of a conventional photonic waveguide, the TIR mechanism acts in the *x* and *z*-directions. For the implemented SSH device with a domain wall in the *x*-direction, the topological boundary state is generated in the band gap split occurring in two band states owing to the binary basis in the crystal unit cell. The confined topological state in the band gap has the advantage that the state is immune to fabrication induced defects in that direction as long as the chiral symmetry is not broken by the defects. In particular, disorder in coupling coefficients do not break the chiral symmetry. We can see that light is well guided even in the presence of severe defects as long as chiral symmetry is preserved.

In this paper, there are two key metrics we achieved. One is efficient FWM conversion efficiency which is comparable to those from other CMOS-compatible platforms. This improvement stems from the high linear and nonlinear refractive index inherent in USRN and negligible two-photon absorption near 1,550 nm of wavelength. In addition, the modal confinement was designed to be high in the device, further strengthening the nonlinear interactions occurring within. Here, the USRN platform is designed with topological SSH structure which gives rise to strong localization of topological mode (amplitude) at the domain wall. It ensures that minimal input optical power dissipates out of the central waveguide, helping strongly concentrate the optical field within the central waveguide to generate efficient four-wave mixing.

The other key metric in the paper is the robustness of the FWM conversion efficiency. This effect arises because the topological structure facilitates light propagation in the intended direction even in the presence of disorder. The topological SSH structure helps to stabilize FWM generation, and we show in our paper, how even in the presence of 80 % disorder in the fabricated device’s coupling coefficients, FWM conversion efficiency is maintained since the chiral symmetry of the structure is preserved. This robustness against fabrication error is a key advantage of FWM using the topological structure.

The topological device has strong merit of immunity to fabrication defects as shown in the robustness against 80 % of disorder in the fabricated waveguide coupling coefficients. This robustness arises because the light localization originates from chiral symmetry as opposed to total internal reflection or bandgap guiding, chiral symmetry is preserved in the presence of the disorder. This immunity to defects is useful for robust light transmission, nonlinear interactions, and may be applied in the future for robust high-speed data transmission. For long device lengths, the immunity to defects is important because even small distortions induced by defects (e.g. from fabrication) accumulates along the propagation length. In our device, the boundary state is topologically generated in the middle of forbidden band in the binary SSH distribution. The high extents of disorder could make the band structure broad and smear the forbidden band or cause the forbidden barrier to become shallow. The Kerr nonlinearity inherently breaks chiral symmetry, but the boundary state does not disappear and just pushes up the boundary state from the zero mode in the middle of forbidden band. Thus, we could experimentally observe the robustness of the nonlinear four-wave mixing to a remarkable ±80 % randomness in the coupling coefficients.

Due to the unique feature of robust light transport in topological systems, nonlinear frequency conversion has also been demonstrated in various topological structures. Floquet topological photonic insulators have been demonstrated for efficient parametric processes because it enables control of light direction and localization through the lattice which is perturbed by the driving sequence of the system [49], [50], [51], [52], [53]. Recently, resonance enhanced four-wave mixing was demonstrated using a 2D silicon Floquet microring lattice, a type of Floquet topological photonic insulator [52]. While the demonstrated conversion bandwidth is limited, the result is noteworthy since resonance enhancement in the localized Floquet bulk modes enabled a 12.5 dB increase in the conversion efficiency. Ref. [53] reports an all-band flat Floquet–Lieb topological insulator for broadband four-wave mixing, demonstrating conversion bandwidths as high as 50 nm at an input pump power of 1.4 mW. In our work, the maximum conversion bandwidth achieved is 54 nm at an input pump power of 3 mW.

Perhaps one of the most interesting aspects of topological systems is their robustness to various types of disorder. In the context of parametric processes demonstrated to be robust against disorder, Ref. 49 utilizes a zigzag array of nanoresonators for third harmonic generation. The topological system preserves chiral symmetry and the edge state energies, enabling robustness against disorder in the nanoresonator angles. In our work, robustness against disorder in the positions of the waveguides in the SSH array, the magnitude of which quantified as 80 % of the coupling coefficient, was successfully demonstrated through similar FWM conversion efficiencies achieved in disordered and disorder-free SSH devices. In this case, the system’s topology preserves the chiral symmetry even in the presence of fluctuations in the coupling coefficients in the array. This unique attribute makes the device useful for stable, broadband frequency generation in low-power-consumed compact system, further providing a vessel for onward studies of disorder robust nonlinear topological optical phenomena.

In the future, further studies of devices possessing greater extents of disorder and their impact on the device performance may be undertaken. It would be interesting to investigate the robustness of other types of nonlinear phenomena in our topological SSH design. High-speed data transmission in topological SSH structures which contain chiral symmetry conserved defects could also be investigated. The signal impairment induced by parasitic defects is an important problem. For the propagation of high-speed data over long distances, the reduction in the bit error rate is accumulated even in the presence of weak distortions especially when the modulation rate is high. The topological photonic device could be a potential solution.

## 5 Conclusions

In conclusion, we observe wavelength-tunable CW FWM in the boundary waveguide of the USRN SSH topological waveguide. We demonstrate efficient CW FWM at an ultra-low pump power of 0.51 mW. This is the lowest pump power used for CW FWM with continuous wavelength tuning in CMOS-compatible platforms for which an observable idler is generated. In addition, the conversion efficiency of the SSH devices with and without disorder is experimentally characterized to be similar to each other. Our study demonstrates that the USRN SSH waveguide effectively generates CW FWM, robust in the generation of nonlinear conversion even in the presence of 80 % randomness in the coupling coefficients within the array. These unique attributes may enable future implementations of low power, compact and tunable systems robust against fabrication imperfections, for wavelength conversion, parametric amplification, and signal regeneration.
